# **Critical to Quality Assessment Methodology: An Adaptable Clinical Trial Risk Management and Quality Assurance Approach for Quality Functions in Pharmaceutical Companies, Developed by the Inter-Company IMPALA Consortium**

**DOI:** 10.1007/s43441-025-00892-x

**Published:** 2026-05-11

**Authors:** Kiernan Trevett, Jennifer Emerson, Paula Walker, Timothé Ménard, Mariza Koukounari, Louise Anderson, Heike Barlow, Jamie Bridges

**Affiliations:** 1 Genentech, A Member of the Roche Company, South San Francisco, 94080 USA; 2 Boehringer Ingelheim Pharma GmbH & Co KG, 88397 Biberach an Der Riss, Germany; 3 Roche Products Ltd, Welwyn Garden City, AL7 1TW UK; 4 F. Hoffmann-La Roche AG, 4070 Basel, Switzerland; 5 Bristol Myers Squibb Pharmaceuticals Ltd, Uxbridge, UB8 1DH UK; 6 Bayer Plc, Reading, RG2 6AD UK; 7 Merck & Co Inc., Rahway, NJ 07065 USA

**Keywords:** Clinical trials, Critical to quality, Risk-based quality management, Quality assurance, Data analytics, Critical to Quality (CtQ), Good Clinical Practice (GCP)

## Abstract

Clinical trial regulations recommend a dynamic and risk-based approach to the quality management strategy for a medicinal product development program. Sponsor companies are required to identify factors that are critical to quality, to perform ongoing evaluation of the changing risks and quality status in clinical trials and adapt their quality strategy accordingly. The Inter-Company Quality Analytics (IMPALA) not for profit consortium supports the shift towards dynamic quality management of clinical trials by providing publicly available data analytics packages to measure changing risks to patient safety and data reliability. The Critical to Quality Assessment Methodology is the latest initiative developed by the IMPALA consortium in support of dynamic quality assurance and risk management. This paper describes the methodology, which is an iterative process conducted throughout a clinical trial, beginning with risk factor identification and assessment, and development of the initial quality strategy based on this evaluation. It continues with the generation of quality evidence through the execution of a variety of activities, including quality assurance audits, process compliance monitoring, real-time study surveillance activities, etc., utilizing both traditional and analytical methods. From there quality conclusions are determined and documented in the Critical to Quality Assessment Report (CAR). These steps are executed on a continuous basis throughout the development of a medicinal product across all clinical trials and the CAR serves as a pivotal knowledge management tool, disclosing Good Clinical Practice compliance conclusions for stakeholders.

## Introduction

Over the past 10 years, there has been a steady shift in clinical trial-related regulations recommending a more dynamic approach to the quality management strategy for a medicinal product development program. Rather than establishing a fixed quality strategy executed the same way for every clinical trial, the regulations have encouraged sponsor companies to adapt the quality strategy based on the characteristics of each clinical trial and the changing circumstances which develop during clinical trial execution [1–3]. This has led sponsor companies to investigate the adoption of more dynamic strategic approaches to managing quality in clinical trials. The *Reflection paper on risk based quality management in clinical trials* (2013) [1] from the European Medicines Agency (EMA) acknowledges that the current practices to achieve quality in clinical trials are neither proportionate nor scalable; in other words, perfect quality may come at an unnecessarily high cost or will fail, which is also costly. For this reason, they advocate a risk-based approach to managing quality in clinical trials. A more recent change in the regulations that has formed the cornerstone of this shift towards dynamic quality management is the revision to the *General Considerations for Clinical Studies* (ICH E8 R1) [2]. The revision incorporates the concept that the quality management approach in clinical trials should be fit for purpose and align with the study intent and design. This is achieved by identifying and implementing quality by design (QbD) principles during the design phase of the clinical trial. Effective QbD begins with identification of factors that are critical to quality [3], which are defined as specific aspects or characteristics of the trial that directly impact the quality, integrity and validity of trial data, as well as patient safety and regulatory compliance. These factors are essential for ensuring the overall success of the clinical trial. The changes to ICH E6 *Good Clinical Practice* with the release of Revision 3 (R3) further supports the move towards a dynamic approach to developing and implementing the quality management strategy for clinical trials. Section 3.10 *Quality Management* of ICH E6 (R3) [4] outlines that activities to support quality in clinical trials should be based on CtQ factors and risks, and that the quality strategy should be dynamic to represent changing risks to patient safety and data reliability. Therefore, an ongoing evaluation of evolving risks supports quality strategy decision-making. While CtQ factors may focus on operational risks, it’s important to note that risk-based quality management (RBQM) is not limited to operational activities but also extends to Quality Assurance (QA) activities. By integrating RBQM principles broadly into quality processes, organizations can optimize resource allocation, ensure activities are aligned with the risk landscape of the trial, thoroughly evaluate and mitigate potential risks, and strengthen overall trial quality and compliance.In response to the changes in the clinical trial regulatory landscape, Quality Management leadership in some sponsor companies have adopted a more dynamic approach to defining and executing the quality strategy, including measures to assure quality and compliance and manage risk. The limitations of traditional QA programs are recognized, including the inherent retrospective nature of the activities, coupled with the relatively small dataset included in audit samples and the often high degree of manual review of data and documentation. The IMPALA (Inter-Company Quality Analytics) not-for-profit consortium is an important contributor to the shift towards a more comprehensive approach to managing quality in clinical trials by developing and releasing data analytics packages that generate insights when applied to incoming clinical trial data that are critical to decision making. Examples of IMPALA-developed data analytics packages include {simsaerep} and the Clinical Trial Anomaly Spotter (CTAS). Both packages target the detection of data anomalies which could indicate changes in quality status. The {simsaerep} data analytics package was initially developed and deployed within one of the IMPALA member companies [5, 6] for the detection of potential adverse event under-reporting by clinical trial sites. This package was subsequently implemented and validated by other IMPALA member companies seeking support with evaluating the changing quality status of clinical trials [7]. The main focus of the CTAS package is on flagging sites with one or more study parameters whose profiles differ from those of the other sites and identifying anomalies for individual subjects [8]. The next step in the evolution of IMPALA work products is the CtQ Assessment Methodology, including the Critical to Quality Assessment Report (CAR). The methodology is grounded in QbD, CtQ and RBQM principles, and is a process of assessing risks, developing a quality strategy and collecting evidence to support the generation of a quality conclusion. This process is executed on a continuous basis throughout a clinical trial recognizing that, as the study evolves, so too does the quality strategy. The CAR represents the RBQM output for the development program, providing a meaningful summary of RBQM effectiveness throughout the life cycle. It also supports proactive transparency to regulatory authorities (RA) on development issues, including their mitigation and impact upon clinical data reliability and patient safety. The IMPALA consortium sees an opportunity for the R&D Quality function to be an enabler in accelerating the review and approval processes for new medicinal products by sharing GCP compliance evidence with regulators on a rolling basis, rather than retroactively at the point of application filing. A tool such as the CAR represents a consolidated, practical artifact that enhances regulatory oversight and supports risk-based decision-making by RAs, including in relation to the scope and duration of pre-approval inspections. IMPALA’s aim is to strengthen the inspection process by making compliance information more accessible and usable, and to help inspectors focus on meaningful areas of oversight, deepening the value of inspections without changing their authority or purpose.

### Critical to Quality Assessment Methodology

**Fig. 1:**
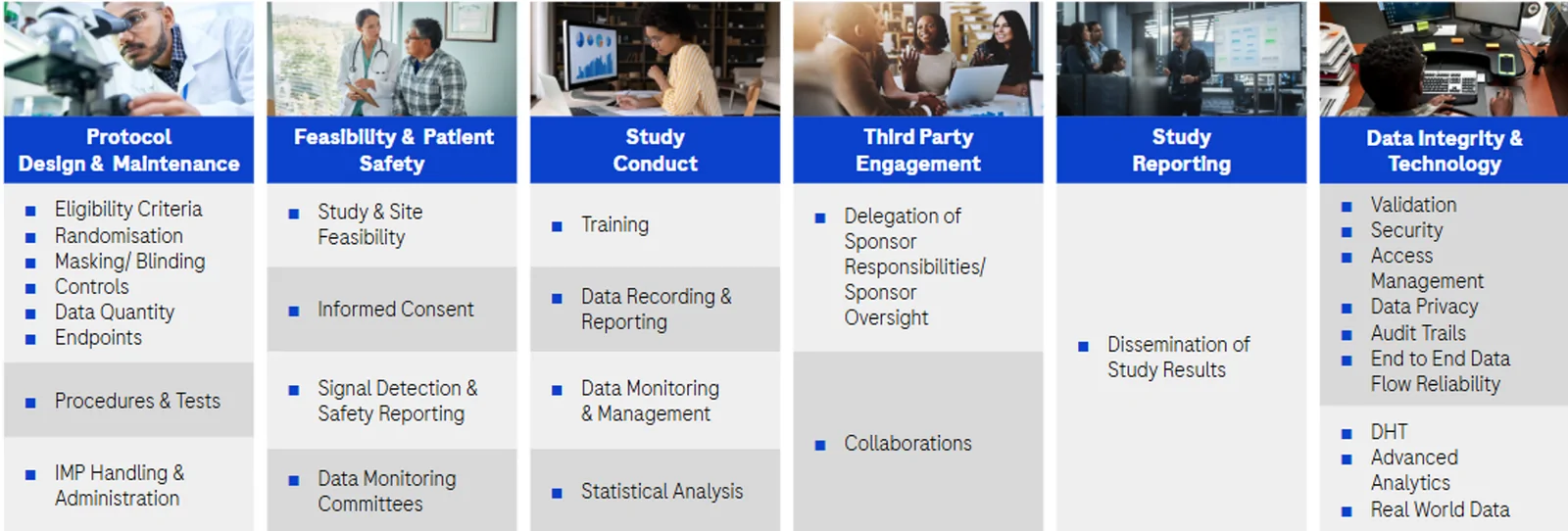
IMPALA critical to quality factors for clinical trials

**Fig. 2:**
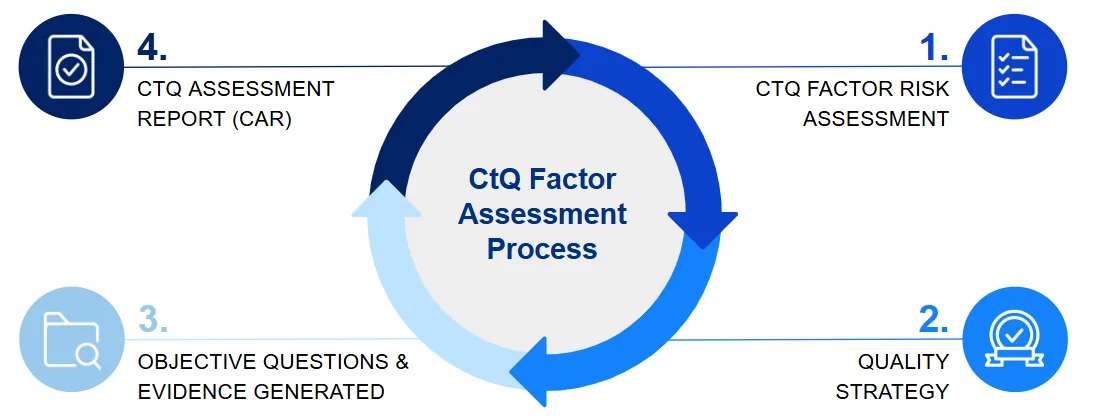
CtQ assessment methodology

The foundation of this methodology is an embedded CtQ framework that encompasses QbD, RBQM and QA, ensuring that activities to support quality in clinical trials are aligned and anchored on CtQ factors and their risks. The Clinical Trials Transformation Initiative (CTTI) developed a CtQ framework that is intended to support proactive, cross-functional discussions and decision-making at the time of trial development about 1) what aspects of a trial are critical to generating reliable data and providing appropriate protection of research participants (CtQ factors) and 2) what strategies and actions will effectively and efficiently support quality in these critical areas [9]. The IMPALA consortium developed a list of CtQ factors that is heavily based on the published CTTI framework (Fig. 1), taking the opportunity to update the list to reflect emerging critical areas that impact upon the clinical development process, such as digital health technologies and artificial intelligence. Effective collaboration between the Clinical Quality function and study teams is pivotal in defining the CtQ factors for the trial and identifying risks that may threaten the integrity of those factors, along with their associated control strategies. In keeping with the guidance of ICH E8 (R1), emphasis should be given to those factors that are relevant to ensuring study quality, particularly those factors that are connected with protection of study participants, the reliability and interpretability the study results, and the decisions made based on the study results [2]. For example, where endpoints have a clinically meaningful impact on the continuation of patient treatment in oncology trials, factors related to the reliability of endpoint data become critical. Methodologies used in endpoint assessment, such as central blinded reading, along with the established criteria for adjudication, are essential to consider for effectively anticipating risks associated with verifying endpoint outcomes like disease progression. Additionally, it may be important to evaluate CtQ factors and associated risks related to investigational product handling and administration, particularly if the protocol specifies dosage adjustments for the investigational product or control product in response to toxicities. Clear directions and procedures for making these dosage adjustments, as well as clearly defined responsibilities, are crucial for ensuring compliance and participant safety. Addressing CtQ factors related to blinding in protocols, particularly in scenarios where certain site staff members must be unmasked while others remain masked, is also important. Clearly delineating the responsibilities among site personnel and anticipating potential situations that could lead to unintentional unmasking is crucial for minimizing bias in data interpretation. Furthermore, consideration of CtQ factors related to eligibility is necessary, especially when specific criteria are critical to trial participant evaluability or safety (e.g., contraindicated medications or prior treatments). Figure 2 outlines the minimum viable elements of the Critical to Quality Assessment Methodology, which is intended to be a continuous process taking place throughout a clinical trial. The following sub-sections of this article describe each of the steps in the process. It is recognized that sponsor companies will adapt the methodology and its output according to their specific circumstances.

### CtQ Factor Risk Assessment

Once the CtQ factors are defined, and ranges or tolerance limits are established, the corresponding risk factors are assessed for each CtQ factor. This enables the sponsor to identify risks that may have a meaningful impact on the integrity of the CtQ factors. Based on the risk assessment, risk mitigation activities may be incorporated in protocol design and implementation, monitoring plans, systematic safeguards, etc. The implementation of these risk mitigation activities is typically performed by operational study teams; however, the role of the R&D Quality function as a cross-functional enabler of effective QbD and RBQM should not be underestimated. Quality professionals can guide cross-functional study teams through study design to minimize unnecessary complexity and burden, incorporating learnings from inspection findings and disease area risk mitigation strategies. A strong focus on CtQ factors ensures that non-critical data review is removed, freeing up vital resources to safeguard critical data and processes. Quality assurance activities are a key requirement of ICH E6 (R3) (Sect. 3.11) and provide independent verification of whether trials are conducted in compliance with the protocol, GCP and applicable regulatory requirements. In the context of this methodology, quality assurance activities provide independent evidence of whether CtQ factor risks have been effectively managed. Quality professionals perform ongoing risk analysis to identify emerging threats to CtQ factors and develop quality strategies to evaluate the effectiveness of risk control strategies and generate evidence of compliance. The frequency of ongoing risk analysis for each trial should consider the importance of the trial to submissions to RAs, the number of participants in the trial, the type and complexity of the trial, the level of risks to the trial participants and any identified problem duration. Data should be leveraged in the most effective way to generate insights that can allow for meaningful risk identification and characterization. The inclusion of data analytics packages as a central component of risk management underscores the significance of leveraging open-source, collaborative efforts to advance practices in the realm of R&D Quality. Key components for effective implementation of data analytics for risk management include the following elements. First, there should be a pre-defined methodology to determine the measurable quality features for the specific CtQ factors/processes (e.g. informed consent) across the data landscape. Next, the data outputs should be assessed for signals and insights that may indicate risk and point towards a particular quality intervention, which may include audits, process/document updates, or implementation of systematic safeguards to address identified risks. This should be an iterative process throughout the clinical trial, continually integrating relevant information from multiple sources (e.g. electronic data capture, clinical trial management system) across studies to further characterize CtQ factor risks. All of this taken together results in a risk management process that efficiently identifies emerging risks that threaten the integrity of the CtQ factors and that leads to the development of a tailored quality strategy that drives the generation of evidence to validate process health.

### Quality Strategy

The quality strategy summarizes the plan of proactive quality activities intended to identify potential or actual causes of serious non-compliance with the protocol, GCP and/or applicable regulatory requirements to enable their corrective and/or preventive actions. These evidence-generating activities, including audits, focus on objective questions that are designed to gather the specific evidence needed to draw conclusions on the state of compliance for the CtQ factor. In the context of ICH E6 (R3), the quality strategy is synonymous with the risk-based quality assurance strategy described in Sect. 3.11.1. The list of CtQ factors for clinical trials published by the IMPALA consortium includes examples of objective questions that sponsor companies can use to drive their evidence-generating strategies, focusing on the areas of highest risk for the trial. IMPALA has developed open-source validated data analytics packages that generate compliance insights for interpretation by Quality professionals to support the development of the quality strategy, the selection of objective questions and the delivery of proactive quality activities to generate evidence. The quality strategy is dynamic and should be updated to reflect the outcome of ongoing risk assessment activities and the conclusions drawn based on compliance evidence that has been generated.

### Evidence Generation

The strategic generation and assessment of compliance evidence for the CtQ factors is fundamental to the generation of the quality conclusions in the CAR. Sponsors are required to implement a quality management system to ensure that trials are conducted in compliance with the protocol, GCP and applicable regulatory requirements, and compliance evidence can be derived from quality assurance audits, process compliance monitoring and real-time study surveillance activities, as examples.Traditional audit programs are designed to evaluate different entities across the clinical trial landscape using risk-based selection criteria. The known limitation of traditional audits is the inherent retrospective nature of the activity, coupled with the relatively small dataset included in the audit sample and the often high degree of manual review of data and documentation. Within Clinical Quality there are several areas where data analytics can be applied to support audit risk assessment and to generate evidence of compliance in critical areas. The IMPALA consortium has a mission to advance the use of analytics in the GCP and Good Pharmacovigilance Practice (GVP) environment and has published a framework for guiding Sponsors’ QA functions in the development, implementation and enhancement of their analytics and technology strategy [10]. Open-source analytics packages should be integrated into the framework of QA activities, for example analytics-based audit [6, 11]. QA processes, such as on-site audits and manual source data verification (SDV), have historically relied on limited sampling methods that may not fully capture the complexities and nuances of CtQ aspects, e.g. safety reporting and sponsor oversight, across multiple clinical trials. However, incorporating data analytics packages into the audit process brings forth a comprehensive and data-driven approach. Quality professionals gain the capability to holistically scrutinize an entire program comprising multiple clinical trials, resulting in a fundamental shift in audit methodology that significantly enhances efficiency, accuracy and effectiveness in quality oversight for clinical trials. Open-source analytics packages empower Quality professionals to swiftly and efficiently analyze vast amounts of data. Utilizing advanced data analytics techniques, potential issues or anomalies can be identified with greater precision and speed [5, 11]. Furthermore, the comprehensive approach enabled by the analytics package ensures more repeatable and consistent quality oversight, reducing the likelihood of overlooking critical issues during audits.In addition to enhancing accuracy and efficiency, the integration of the analytics package into the audit process plays a pivotal role in QA evidence generation. QA evidence encompasses the data, insights and findings acquired during the audit, which can be transparently shared in a CAR. This evidence is derived from the comprehensive analysis of large datasets, allowing Quality professionals to identify patterns, trends and potential deviations from established quality standards. Moreover, the evidence generated through these audits can be shared transparently, facilitating knowledge sharing and continuous improvement within the industry. A practical application of this is the use of a data-driven, analytics-based audit methodology: Real-time Audit Package Informed by Data (RAPID) [12]. This approach follows a pre-defined methodology to assess specific CtQ processes, such as informed consent, and efficiently identify systemic issues. For evidence generation, a dedicated data analytics package (DAP) is deployed to analyze data at scale, providing a fast and holistic view by integrating information from multiple sources (e.g., electronic data capture, clinical trial management system) across studies. The DAP is designed to programmatically generate outputs against pre-defined objective questions, such as verifying that informed consent was obtained prior to any study-related procedures. This enables a more efficient audit conduct, allowing for assessment at scale. The resulting QA evidence is a synthesis of the insights generated by the DAP combined with the auditors’ domain expertise. This combination allows them to efficiently validate potential systemic issues and, importantly, to confirm positive affirmations of process health. This data-driven approach allows audit teams to move beyond limited sampling, generating robust QA evidence for the CtQ factor in a fast, holistic and efficient manner.

### Critical to Quality Assessment Report

The purpose of the CAR is to present a robust and meaningful appraisal of the GCP compliance status of the CtQ factors for clinical trials for medicinal products in order to monitor their integrity on an ongoing basis and support quality interventions, where needed. The CAR includes a succinct summary of the CtQ factors for the trial(s), along with a description of their associated risks and risk control strategies. It then presents the evidence obtained from the quality strategy that demonstrates whether the risks were adequately controlled. Importantly, this evidence includes positive affirmations, where these were identified as part of the evidence-generating activities, which demonstrate that processes are in a state of control. The CAR also includes a summary of important quality issues and describes the impact of identified deficiencies in the context of study data reliability and/or the safety of research participants. Based on the quality evidence generated through the execution of the quality strategy, a conclusion on the state of compliance for the CtQ factor can be determined. The following is an example of how the compliance conclusion could be expressed:

#### CtQ Factor is in a State of Control

The evidence gathered through quality assurance activities demonstrates that the Critical to Quality Factor is in a state of control. It indicates that the organization has implemented robust processes and controls that protect the rights, safety and well-being of research subjects and patients and ensure the reliability of data. The favorable outcome signifies that any identified issues, although present, either have a low impact on data integrity and/or patient safety or the impact has been adequately addressed through appropriate corrective and preventive actions.

#### Control Strategies Planned/Ongoing

The evidence gathered through quality assurance activities demonstrates that issues in relation to the Critical to Quality Factor have been identified and there is a potential impact on data integrity and/or patient safety. Due to the risk to the Critical to Quality Factor, control strategies are being implemented to limit the impact of the identified issues and a robust monitoring mechanism is in place to ensure their effective implementation.

Such conclusions provide transparency about the reliability of data collected during the trials, adherence to defined study processes, oversight of the vendors involved in product development activities and suitability of computerized systems used during the product development life cycle. Based on the conclusions, strategic quality interventions, such as continuous process improvement initiatives, risk mitigation actions and quality assurance audits, are proposed to manage risks and obtain further compliance evidence. The methodology allows sponsor companies to document the compliance status of the CtQ factors in the CAR on an ongoing basis throughout the product development life cycle and share this with key internal stakeholders, as well as with RAs at various milestones, including marketing authorisation application (MAA) submission and pre-approval inspections. Having this information in a single location serves as an important historical record and source for institutional learning throughout a compound’s development. The CAR serves as the knowledge management vehicle for disclosure of compliance conclusions for CtQ factors to internal stakeholders, and also supports proactive transparency to RAs on development issues, including their mitigation and impact upon clinical data reliability and patient safety.

## Discussion

The CtQ assessment methodology consists of generating compliance evidence from proactive quality activities, focusing on the CtQ factors for the study and the risks related to essential safety and efficacy data. Evidence can be derived from quality assurance audits, process compliance monitoring, real-time study surveillance activities, etc., leveraging data analytics solutions for clinical quality wherever possible. This compliance evidence is evaluated by the sponsor’s Quality function on an ongoing basis to draw conclusions on the state of compliance for the CtQ factors. The shift to real-time compliance evaluation of the CtQ factors for the trial enables the Quality function to partner more effectively with the study teams to manage risks and drive quality outcomes. As the CAR methodology encompasses ongoing generation and review of compliance evidence, the results of process improvement activities can be continuously evaluated and assessed for their effectiveness in resolving issues and mitigating impact. In effect, quality assurance becomes more dynamic, allowing sponsor companies to respond to quality risk signals in real time and adapt their approaches to continuously ensure patient safety and data integrity. This article has presented the CAR methodology in the context of the relevant ICH guidelines e.g. ICH E6 (R3) and ICH E8 (R1); however, the methodology is also grounded more broadly in the regulations related to the Pharmaceutical Quality System. ICH Q8 (R2) provides guidance for Pharmaceutical Development for drug products and includes the concept of the Quality Target Product Profile (QTPP), which considers the intended use of the compound and the unique critical quality attributes of a drug product. In a similar way, the CAR methodology begins with the CtQ factors as the central component, which are tailored to the unique characteristics of the clinical trial or product development plan. Furthermore, the ICH Q8 (R2) framework guides users to perform risk assessments and design a strategy based on the output of these activities. Grounding the CAR methodology not only in ICH E6 and E8 but also in Q8 strengthens the rationality of the approach. The CAR methodology has multiple benefits for biotechnology and pharmaceutical companies. The methodology foresees that there is a focus on outcomes rather than traditional outputs and, because of this, risks are identified and properly mitigated proactively, which may result in prevention of issue development. In case issues do develop, they are detected early through the regular cycle of review and assessment and their impact is assessed in real time, acting as an enabling methodology supporting clinical development. The role of Quality professionals is becoming increasingly essential in driving quality-related innovations, such as the development of CARs. As technology advances and healthcare regulations continue to evolve, the skillset required of Quality professionals must also adapt. In addition to traditional competencies, new capabilities such as proficiency in digital tools, data analysis and the implementation of risk-based strategies will be essential. These skills are not only necessary for crafting evidence-based conclusions within CARs but also for supporting these conclusions through insights derived from data analytics. Moreover, the ability to complement findings with positive affirmations of compliance requires critical thinking and a nuanced understanding of regulatory expectations. Therefore, training and development programs that emphasize digital proficiency, analytical skills, risk management and regulatory knowledge will be crucial in preparing Quality professionals for the demands of contemporary quality management. In leading the CAR process, Quality professionals will need to collaborate closely with operational teams within the RBQM framework. This partnership will ensure that all available study surveillance activities and outcomes are integrated into the CAR’s compliance conclusions. To address any gaps in evidence, a flexible audit strategy will be necessary to enable the conduct of targeted compliance audits based on identified risks, quality incident data and inspection outcomes. Successful implementation of the CAR will depend on interdisciplinary collaboration, continuous skill development and staying current with industry trends. By embracing these practices, Quality professionals can shift quality management from a reactive to a proactive approach, leading the way in modernizing quality assurance practices and enhancing the overall quality landscape.

## Regulatory Authority Engagement

An intended benefit of the methodology is that the transparency inherent in sharing the CAR with RAs should build trust and confidence in the compliance status throughout the product development life cycle. Through this proactive transparency, the intention is to enable the regulatory assessment process, including risk-based inspection scope, eventually reducing late-stage issue discovery as part of pre-filing or pre-inspection quality assurance preparation, and prevent delays in approvals. Ultimately this supports trust building and confidence with RAs, getting products through to patients as quickly as possible while maintaining rigorous quality and safety standards. At the time of writing, several examples of CARs (formerly termed Quality Briefs) have been submitted to RAs in the US and Europe in filing submission packages. The IMPALA consortium is actively engaging with RAs on the CAR methodology and value proposition, with a view to developing an output that meets the needs of the RAs and that can be validated during regulatory inspections. The US FDA participated in a session at DIA Global 2025 where a representative from the Office of Scientific Investigations indicated that the CAR approach has the potential to enable QbD by identifying CtQ factors and associated risks, and providing a practical iterative framework applicable throughout the clinical trial lifecycle. The discussion during the session alluded to an opportunity for the CAR to increase transparency in quality expectations and CtQ factor/risk identification and help align stakeholders around prioritized CtQ factors. The IMPALA representatives in the session emphasized the potential role of the CAR in strengthening communication with regulators through clear articulation of CtQ factors and their associated risks. Further engagement with the FDA will be sought to explore practical pathways to pilot the CAR approach in the US more formally. Early engagement with regulators in Europe, including through an EMA Innovation Task Force briefing meeting, has also yielded variable and valuable feedback on the CAR methodology and value proposition. One key concern raised was regarding maintaining the independence of the Quality Assurance function, as ICH E6 R2 and R3 both emphasise the need to preserve the independence and value of the audit function by RAs not routinely requesting audit reports. IMPALA’s position is that the CAR is under the responsibility of the Quality function, ensuring independent oversight of trial quality and compliance, thereby safeguarding the independent nature of the methodology. There is also a regulatory precedent for disclosure of audit findings to regulators through the introduction in 2012 of the requirement for marketing authorisation holders to maintain and submit a pharmacovigilance system master file (PSMF) for their EU authorisations. The PSMF includes audit findings, along with a summary of the associated corrective and preventative actions. Another concern raised by European regulators is that the CAR is overly data-driven and may overlook the process/system context. RAs noted that not all CtQ factors can be monitored through analytics and some may require alternative monitoring activities, such as conducting an audit of a process if an endpoint is managed by an external committee, or auditing a vendor for ePRO data. IMPALA’s position is that the methodology is designed as an iterative process that focuses on identifying areas where analytics can provide the most benefit, while recognising that some CtQ factors may necessitate different oversight strategies. There were also questions from regulators about how the CAR aligns with the existing requirements for inclusion of compliance data in the clinical study report (CSR). IMPALA’s position is that the CAR serves as an important supplement to complement the quality summaries made in the CSR, providing more detailed information on significant quality issues and how they were addressed, and offering a more holistic view of the quality status with the inclusion of evidence that demonstrates compliant execution of processes, and not just issues that have occurred. It is also foreseen that the CAR could be shared with regulators on a rolling basis at specific milestones along the study life cycle, rather than retroactively at the point of application filing. In addition, concerns raised regarding the completeness and accuracy of the CAR are well understood and sponsor companies recognize the need to provide high quality, transparent clinical trial quality data and information with full integrity so that RAs can fulfill their mission with confidence and patients can gain timely and safe access to the therapies they need.

## Conclusion

Sponsor pharmaceutical companies are mandated by ICH E8 (R1) and ICH E6 (R3) guidelines to integrate QbD and RBQM approaches into trial design and execution. In alignment with these regulatory requirements, the CTTI has developed a CtQ factor framework to facilitate proactive, cross-functional discussions and decision-making during trial development. The IMPALA consortium has developed a list of CtQ factors that is heavily based on the published CTTI framework, which includes examples of objective questions that Sponsor companies can use to drive their evidence-generating strategies, focusing on the areas of highest risk for the trial. The next step in the evolution of IMPALA work products is the CtQ Assessment Methodology including the CAR, which serves as a pivotal knowledge management tool, disclosing compliance conclusions for CtQ factors to both internal and external stakeholders and elucidating the impact of identified deficiencies on study data reliability and participant safety. The CtQ Assessment Methodology is an aspirational approach to risk-based quality management and is intended to be adapted by Sponsor companies according to their specific circumstances.

Quality professionals must collaborate closely with operational teams within the RBQM framework to lead the CAR process. This partnership will ensure that study surveillance activities, including the use of data analytics wherever possible, are integrated into the CAR’s compliance conclusions. The long-term goal of the CAR strategy is to expedite the delivery of new products to patients while providing RAs with efficient, real-time access to information on compliance and RBQM effectiveness; crucial for inspection and approval outcomes. Several CARs have already been submitted to RAs in the US and Europe as part of filing submission packages. The IMPALA consortium is actively engaging with these authorities to refine the CAR format and content, aiming to develop an output that meets regulatory needs and can be validated during inspections. Based on this feedback, the IMPALA consortium intends to produce publicly available tools to support Sponsor companies with the implementation of the CtQ Assessment Methodology and the CAR in due course.

## Data Availability

No datasets were generated or analysed during the current study.
